# Predicting the Temperature-Dependent Long-Term Creep Mechanical Response of Silica Sand-Textured Geomembrane Interfaces Based on Physical Tests and Machine Learning Techniques

**DOI:** 10.3390/ma16186144

**Published:** 2023-09-10

**Authors:** Zhiming Chao, Haoyu Wang, Hanwen Hu, Tianchen Ding, Ye Zhang

**Affiliations:** 1 Shanghai Estuarine and Coastal Science Research Center, Shanghai 201201, China; 2 Institute of Water Sciences and Technology, Hohai University, Nanjing 211106, China; 3 College of Ocean Science and Engineering, Shanghai Maritime University, Shanghai 200135, China; 4 Mentverse Ltd., 25 Cabot Square, Canary Wharf, London E14 4QZ, UK

**Keywords:** sand, creep shear mechanical response, SVM, BPANN

## Abstract

Preciously assessing the creep mechanical response of sand–geomembrane interfaces is vital for the design of relevant engineering applications, which is inevitable to be influenced by temperature and stress statuses. In this paper, based on the self-developed temperature-controlled large interface shear apparatus, a series of long-term creep shear tests on textured geomembrane–silica sand interfaces in different temperatures, normal pressure, and creep shear pressure were conducted, and a database compiled from the physical creep shear test results is constructed. By adopting the database, three disparate machine learning algorithms of the Back Propagation Artificial Neural Network (BPANN), the Support Vector Machine (SVM) and the Extreme Learning Machine (ELM) were adopted to assess the long-term creep mechanical properties of sand–geomembrane interfaces while also considering the influence of temperature. Then, the forecasting results of the different algorithms was compared and analyzed. Furthermore, by using the optimal machine learning model, sensitivity analysis was carried out. The research indicated that the BPANN model has the best forecasting performance according to the statistics criteria of the Root-Mean-Square Error, the Correlation Coefficient, Wilmot’s Index of Agreement, and the Mean Absolute Percentage Error among the developed models. Temperature is the most important influence factor on the creep interface mechanical properties, followed with time. The research findings can support the operating safety of the related engineering facilities installed with the geomembrane.

## 1. Introduction

As an excellent water-proofing material, geomembrane is extensively used as construction material in various engineering applications [1,2,3,4,5]. With the development of a geomembrane manufacturing technology, different types of this material are available. Among them, textured geomembrane is the most popular in practical engineering applications because textured geomembrane can form a strong interface mechanical performance when in contact with other construction material, such as soil, due to the existence of texture on the geomembrane surfaces [6,7]. Although compared to other types of geomembrane, the interfaces between textured geomembrane and soil have better stability, but the textured geomembrane–soil interface is still the weakest component in engineering facilities [8,9,10,11,12,13]. Thus, the correct estimation of the interface mechanical properties between textured geomembrane and soil is critical for the operating safety of relative engineering facilities. 

In general, the service life of geomembrane in engineering infrastructure is over decades [14,15]. During the operation of engineering applications, the textured geomembrane–soil interface is often subjected to constant creep shear stress resulting from the overlaying construction material [16,17]. The long-term creep interface deformation has an obvious difference with the short-term deformation caused by the rapid loading of shear stress. The occurrence of creep shear deformation is more hidden and the deformation can rise rapidly in a short period, which has a more significant hazard on the safety of engineering facilities [18,19]. It highlights the necessity of preciously predicting the long-term creep mechanical response of textured geomembrane–soil interfaces. 

In reality, the engineering environment is complex, and the buildings installed with geomembrane are often subjected to temperature loadings [20,21,22,23,24,25]. For example, due to the exothermal reaction of buried waste biodegradation, the inside temperature of landfills can reach 60~80 °C [26,27]. As a common water-proofing material, it is inevitable that geomembrane installed in landfills will experience a high temperature environment. Since the main raw material of producing geomembrane is thermal-softening plastic materials such as nylon, polyethylene, etc., in elevated temperatures, the softening of geomembrane may occur [28]. This has an obvious influence on the interface mechanical properties between geomembrane and soil [16]. Especially for textured geomembrane, the softening of texture can remarkably change the interaction between geomembrane and soil to result in a considerable impact on the interface’s mechanical response [29,30]. However, due to the limitation of the temperature-controlled interface shear apparatus, at present, the research involving the assessment of the temperature-dependent mechanical properties for geomembrane–soil interfaces is rare, let alone the investigation about the long-term creep mechanical behavior of textured temperature–soil interfaces by considering the temperature effects. 

The interaction between textured geomembrane and soil is complicated, which leads to the multiple influence factors on the mechanical performance of textured geomembrane–soil interfaces [31,32,33]. Especially for the temperature-dependent creep shear mechanical response, it requires to consider the factors of time and temperature, which further increases the complexity of the interface action mechanism. The complex interaction and multiple impact factors cause the difficulty of predictive modelling for the long-term creep mechanical behavior of textured geomembrane–soil interfaces by considering the temperature effects and adopting the traditional estimation methods, such as the mathematical statistical approach, etc. [15,34]. Due to the development in computer technology, in recent years, the machine learning techniques are extensively adopted to replicate the complex action mechanism by considering multiple influence factors [30,35,36,37,38,39,40,41]. In the field of civil engineering, machine learning modeling techniques have found widespread use, including but not limited to the following:

(1) estimating rock permeability [42,43];
(2) predicting interface shear strength [44,45,46];
(3) assessing cement mortar permeability [47,48].

The existing research manifesting the machine learning methods can describe the complex relationships between plentiful factors with high precision and efficiency. However, in the published research, the investigation involving the modelling of the temperature-dependent long-term creep mechanical response of the textured geomembrane–soil interface by adopting machine learning techniques is not reported. 

In this paper, the effectiveness of the three disparate machine learning algorithms in assessing the temperature–dependent long-term creep interface mechanical response between textured geomembrane and silica sand are compared and analyzed, including the Backpropagation Artificial Neural Network (BPANN), the Support Vector Machine (SVM), and the Extreme Learning Machine (ELM). Moreover, by using the optimal machine learning predictive model, the sensitivity analysis of different influence factors on the creep shear mechanical response of textured geomembrane–silica sand interfaces is conducted to determine the relative importance. The research outcomes can provide an effective tool for estimating the temperature-dependent long-term creep mechanical properties of textured geomembrane–sand interfaces, which can provide a reference for the construction of related engineering services. 

## 2. Physical Experiment

**Figure 1:**
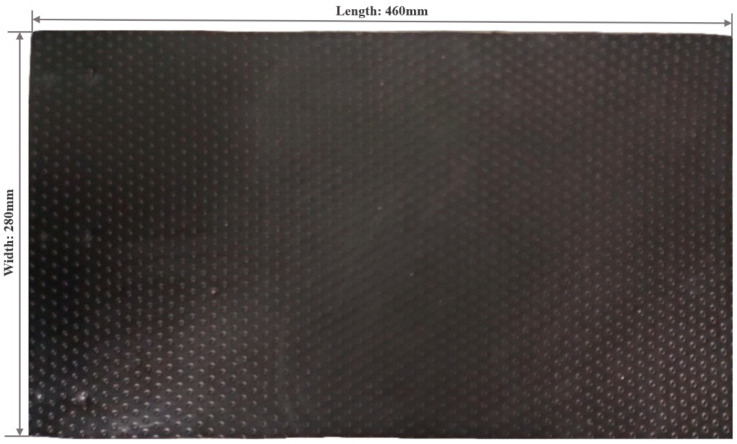
The tested textured geomembrane.

To obtain the data for machine learning modeling, a self-developed large interface direct shear apparatus was utilized, and the tested geomembrane is shown in Figure 1. The temperature–controlled creep shear tests on silica sand–textured geomembrane interfaces were conducted at 25 kPa, 50 kPa, and 100 kPa under normal stress, and the creep shear stress level was 50%, 70%, and 90% of the monotonic peak shear strength for the interface under the corresponding normal stress. For each test condition, three different temperatures of 30 °C, 60 °C, and 200 °C were adopted. The specific test procedure is as follows:

(1) Extured geomembrane and silica sand sample was installed;
(2) The interface temperature was adjusted to the predetermined value and kept stable during the whole test;
(3) The interface was consolidated under the corresponding normal stress for 12 h;
(4) A certain shear stress was imposed on the interfaces and kept stable during the whole test;
(5) The test was terminated until the interface failed or the test duration (6 days) ran out.

The properties of the test material are listed in Table 1, and the test scheme is shown in Table 2.

## 3. Machine Learning Algorithm

By adopting the afore physical test results, three different kinds of machine learning algorithms, BPANN, SVM and ELM, were selected to establish the predictive models on the temperature-dependent creep shear mechanical response of textured geomembrane–sand interfaces by adopting the program software Matlab_R2022b_Windows. The introduction of the adopted machine learning algorithms is presented below.

### 3.1. BPANN

**Figure 2:**
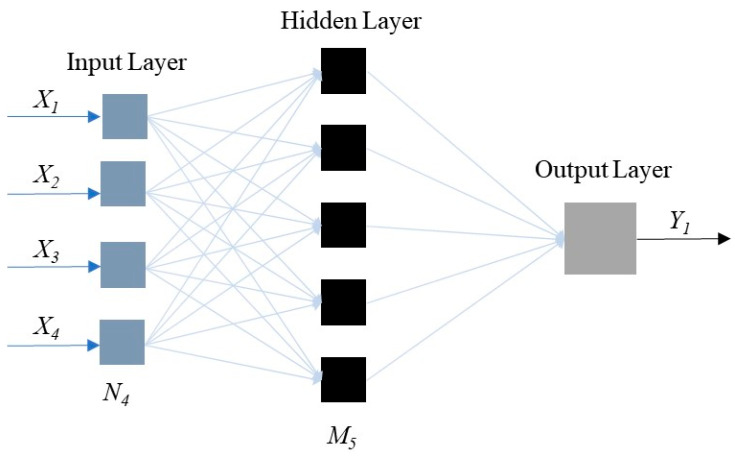
The schematic diagram of BPANN model.

Artificial Neural Network is the most prevalent machine learning algorithm, which can be divided as different kinds [49,50,51]. Among them, the BPANN is the most extensively adopted in civil engineering areas [10,52]. The BPANN model is composed of the input, hidden, and output layers. In this research, the input parameters are normal pressure, creep shear stress, temperature, and time, which corresponds to four joints in the input layer. The output parameter is the creep shear displacement, which corresponds to one joint in the output layer. The joint number of the hidden layer is ascertained by adopting the exhaustive approach, which is 5 in this research. The schematic diagram of the adopted BPANN model structure is shown in Figure 2. The activation function and network training algorithm in the BPANN model adopts the Hyperbolic Tangent Sigmoid Transfer Function and the Levenberg-Marquardt Backpropagation Algorithm, respectively. 

### 3.2. SVM

SVM is another widely adopted machine learning algorithm, which can describe the complex relationship between multiple variables based on the limited number of sample data [53,54]. Also, SVM can project low-dimensional data to high-dimensional data by adopting the kernel function so that the non-linear modelling can be transformed into linear modelling [55,56]. Additionally, SVM can adopt the k-fold cross-validation method (k-CV) in conducting modelling. The theory of k-CV is to divide the original data as equal groups. Among the groups, k-1 groups are adopted as the training data, and the left group is adopted for validation. By selecting different data groups as the testing dataset, the train and validation procedure is repetitive with k times, and the average predictive outcomes of the k times are used as the final value. In this research, the value of k is taken as 10. 

### 3.3. ELM

ELM originates from the ANN model, with the structure being similar with the feedforward ANN [57]. However, in some cases, compared to the ANN model, ELM can achieve complex modelling in a short period, with low computation cost. Like ANN, the joint number of the ELM hidden layer also impacts the forecasting behavior significantly. Therefore, the same exhaustive method is adopted to determine the hidden layer joint number in ELM as 5. For the input and output layer joint number, they are 4 and 1, corresponding to the four input parameters and one output parameter, respectively. The activation function adopts the Logarithmic Sigmoid Function.

## 4. Machine Learning Modelling

### 4.1. Establishment of the Database 

**Figure 3:**
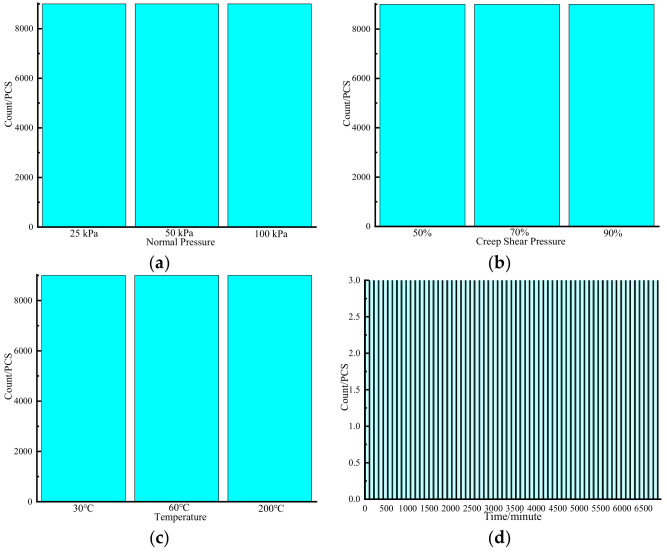
Data distribution of the complied database. (**a**) Normal pressure; (**b**) creep shear pressure; (**c**) temperature; and (**d**) time.

Based on the aforementioned test results, the database was established. The database includes 27,000 datasets. Each dataset consists of four input parameters of normal pressure, creep shear pressure, time, and temperature, and one output parameter of creep shear displacement. The range of normal pressure is from 25 kPa to 100 kPa, and the time is from 0 min to 8640 min, with the temperature ranging from 30 °C to 200 °C. The parameters were chosen because they have large influence on the creep mechanical response of textured geomembrane–soil interfaces, as indicated by the existing research [1,58]. The statistics for the input and output parameters is listed in Table 3. The data distribution for the database is shown in Figure 3, with the *x*-axis and the *y*-axis representing the value of the input parameters and the corresponding data group number, respectively.

### 4.2. Data Processing 

Before machine learning modelling, it was required to divide the database into the training data, to train the models, and the testing data, to validate the predictive performance of the models. In this paper, the division ratio is set as 80% (21,600 groups) for the training data and 20% (5400 groups) for the testing data. Also, to improve the predictive precision and efficiency, the input data were normalized by adopting Equation (1).

(1) $$x_{Normalised}=\frac{2(x-x_{\min})}{x_{\max}-x_{\min}}-1\;$$

where $x_{Normalised}$ and x indicates the normalized and original values, respectively, and $x_{\min}$ and $x_{\max}$ indicates the minimum and maximum values, respectively. 

### 4.3. Precision Assessment Indexes

To quantitatively analyze the forecasting accuracy of the constructed machine learning models, the following four assessment indexes were adopted: Root-Mean-Square Error (RMSE), Coefficient of Determination (R^2^), Mean Absolute Percentage Error (MAPE), and Wilmot’s Index of Agreement (WI), as presented in Equations (2)–(5).

(2) $$\mathrm{RMSE}=\sqrt{\sum_{i=1}^{n}\frac{{\left(y_{i}-f_{i}\right)}^{2}}{n}\;}$$

where *n* indicates the data number, $y_{i}$ indicates the measured data, and $f_{i}$ indicates the assessing value. The less the RMSE value is, the more precise the model.

(3) $$R^{2}=1-\frac{\sum_{i=1}^{n}(y_{i}-f_{i}{)}^{2}}{\sum_{i=1}^{n}(y_{i}-\overset{-}{y}{)}^{2}}\;$$

where $\overset{-}{y}$ indicates the average measured value.

(4) $$\mathrm{MAPE}=\frac{100\%}{n}\sum_{i=1}^{n}\frac{\left|y_{i}-f_{i}\right|}{y_{i}}\;$$

(5) $$\mathrm{WI}=1-\frac{\sum_{i=1}^{n}(y_{i}-f_{i}{)}^{2}}{\sum_{i=1}^{n}(\left|f_{i}-\overset{-}{y}\right|+\left|y_{i}-\overset{-}{y}\right|{)}^{2}}$$

## 5. Result and Analysis 

### 5.1. Determining the Optimal Hidden Layer Joint Number 

**Figure 4:**
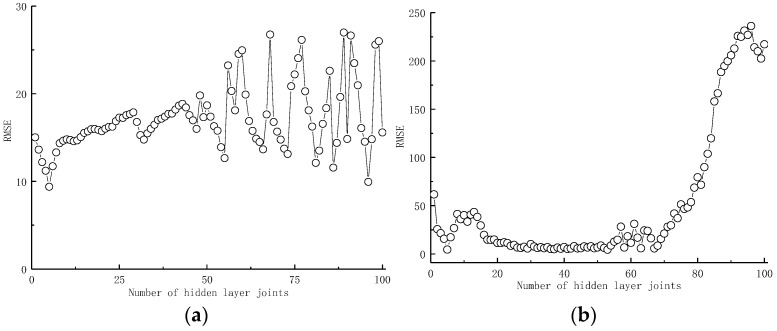
The optimization process. (**a**) BPANN; and (**b**) ELM.

As aforementioned, the hidden layer joint number has a significant influence on the assessing results of the BPANN and ELM models. To determine the optimum number of hidden layer joints, the exhaustive approach was adopted with RMSE as the assessment index. The results are shown in Figure 4. 

Based on Figure 4, the hidden layer joint number has a considerable impact on the RMSE value of the BPANN and ELM models, ranging from 4.23 to 26.97 and from 236.24 to 4.51, respectively. By comparing the assessing results of the BPANN and ELM models containing various hidden layer joint numbers, it is found that when the hidden layer joint number is 5, both the BPANN and ELM models reach the high estimation precision of 4.23 and 4.51, respectively. Thus, in this research, both the BPANN and ELM models adopt five hidden layer joints. 

### 5.2. Assessment Results of Various Machine Learning Models 

**Figure 5:**
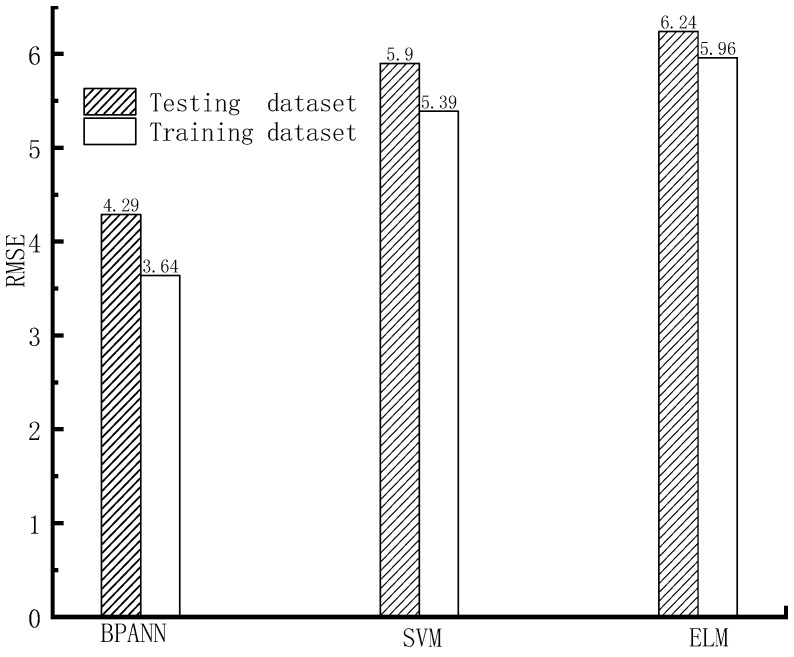
RMSE value.

**Figure 6:**
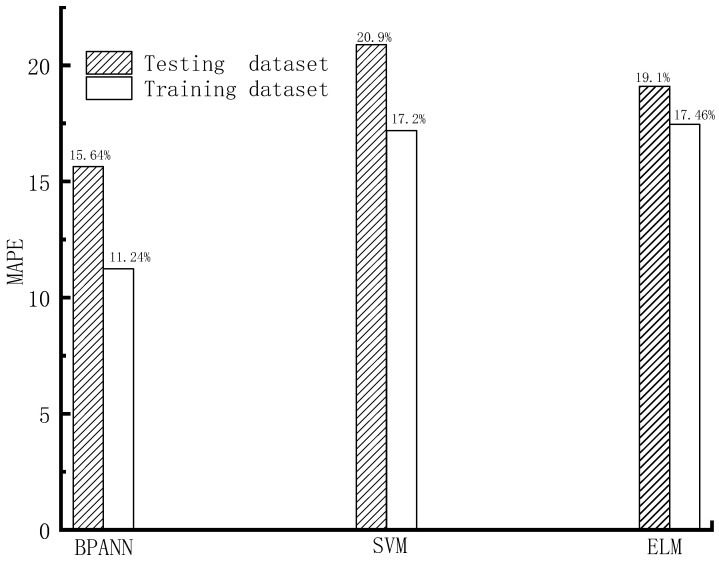
MAPE value.

**Figure 7:**
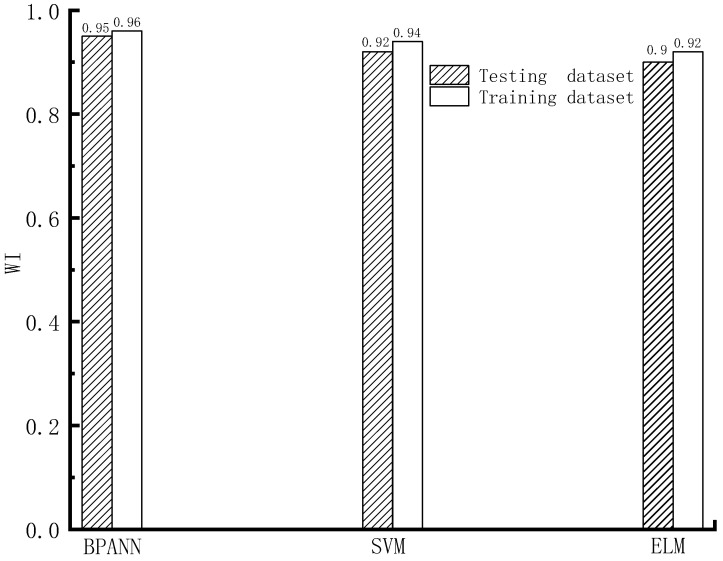
WI value.

**Figure 8:**
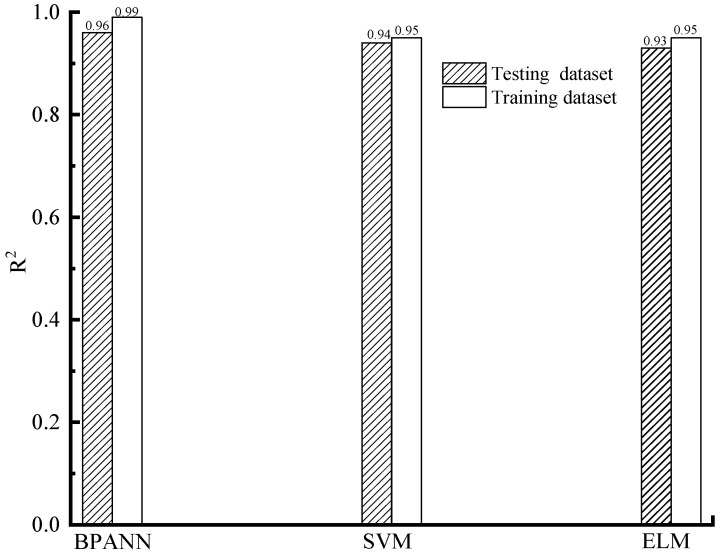
R^2^ value.

The forecasting precision of different machine learning models on the train and test datasets, based on the indexes of RMSE, MAPE, WI, and R^2^, is shown in Figure 5, Figure 6, Figure 7 and Figure 8. 

As shown in Figure 5, Figure 6, Figure 7 and Figure 8, based on the assessment indexes of RMSE, MAPE, WI, and R^2^, the assessing precision of the BPANN model is the highest among the models in the aspect of assessing the training dataset. In the quantitative analysis, the BPANN model achieved the lowest RMSE (3.64) and MAPE (11.24%) and the largest R^2^ (0.99) and WI (0.96). It is followed by the SVM model, and its forecasting performance is better than the ELM model. For example, the RMSE value and the MAPE value of the ELM model (5.96 and 17.46%, respectively) is higher than that of the SVM model (5.39 and 17.2%, respectively).

In the aspect of the forecasting behavior on the testing datasets, as presented in Figure 5, Figure 6, Figure 7 and Figure 8, like the outcomes on the training datasets, the assessing precision of the BPANN model is the best among the different algorithms. More specifically, among the models, the RMSE and MAPE values of the BPANN model is the most low, with 4.29 and 15.64%, respectively, and the R^2^ and WI values of the BPANN model is the largest, with 0.96 and 0.95, respectively. For the SVM and ELM models, their forecasting performance is different based on the different assessment indexes. For example, the RMSE value of the ELM model (6.24) is higher than that of the SVM model (5.9), which indicates the superior performance of the SVM model than the ELM model. In comparison, the MAPE value of the ELM model (19.1%) is less than that of the SVM model (20.9%), which indicates the better precision of the ELM model than that of the SVM model. 

### 5.3. Sensitivity Analysis 

**Figure 9:**
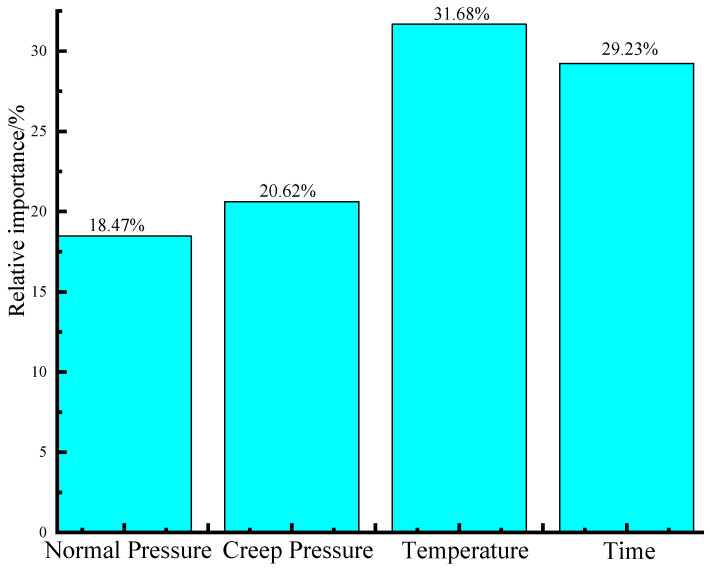
The sensitivity analysis results.

To investigate the relative significance of different factors on influencing the creep shear mechanical response of textured geomembrane–silica sand interfaces, the Garson’s Algorithm was adopted to quantitatively assess the relative significance for the input parameters on the long-term creep shear mechanical properties of the interfaces. The formula for Garson’s Algorithm [59,60] is presented in Equation (6), and the calculated relative significance for the input parameters is depicted in Figure 9.

(6) $$R_{\mathrm{ik}}=\frac{\sum_{j=1}^{L}(\left|W_{ij}W_{jk}\right|/\sum_{r=1}^{N}\left|W_{rj}\right|)}{\sum_{i=1}^{N}\sum_{j=1}^{L}(\left|W_{ij}W_{jk}\right|/\sum_{r=1}^{N}\left|W_{rj}\right|)}$$

where $R_{\mathrm{ik}}$ indicates the parameter relative importance; $W_{ij}$,$W_{jk}$ indicates the connection weight between the input and hidden layers as well as the hidden and output layers; N indicates the input parameter number; and M indicates the output parameter number.

According to Figure 9, temperature has the highest influence on the long-term creep shear mechanical properties of textured geomembrane–sand interfaces, with the relative significance of 31.68%. It is followed by time, with the percentage of 29.23%. In comparison, normal pressure and creep shear pressure have a relatively small impact on the creep shear mechanical response of the interfaces, with the proportions of 18.47% and 20.62%, respectively. 

## 6. Case Study

**Figure 10:**
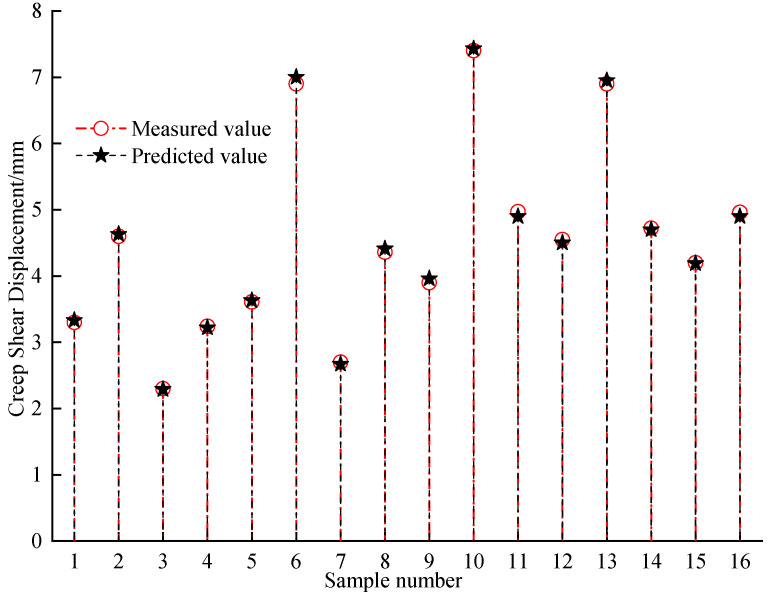
The measured and predicted creep shear displacement.

To verify the reliability of the established machine learning models, a case study is carried out to assess the creep shear displacement of a textured geomembrane–silica sand interface in specific normal pressure, creep shear pressure, temperature, and time by adopting the developed BPANN model. The predicting results were compared with the creep shear displacement measured in the laboratory tests to verify the precision of this model. The specific test conditions and the corresponding input parameters for the BPANN model are listed in Table 4. The predicted creep shear displacement of the interface by using the BPANN model and the measured value in the physical test is presented in Figure 10. 

According to Figure 10, the assessed creep shear displacements of the textured geomembrane–silica sand interface in the specific normal pressure, creep shear pressure, temperature, and time obtained from the BPANN model are similar to the measured creep shear displacements in the physical test. It indicates the developed machine learning model can estimate the temperature-dependent long–term creep shear mechanical response of the textured geomembrane–silica sand interfaces preciously.

## 7. Limitation

Despite the research presenting precious findings, there are limitations for this research. (1) The adopted database for establishing the machine learning models consists of 27,000 datasets, which is not less. However, it still has the room to further enlarge, and the forecasting performance for the machine learning models can be continually enhanced if the bigger database is available; and (2) the influence factors on the creep shear mechanical properties of textured geomembrane–silica sand interfaces are many. Except for the selected input parameters in this research, the properties of textured geomembrane and sand also have impacts on the mechanical response. It should be considered in the future investigation.

## 8. Conclusions

In this paper, by adopting the self-developed large temperature-controlled interface shear apparatus, a series of long-term creep shear tests on textured geomembrane–silica sand interfaces in different temperatures, normal pressure, and creep shear pressure were conducted. Based on the physical test results, the machine learning models for assessing the temperature-dependent long-term creep shear mechanical response of textured geomembrane–silica sand interfaces were established by using three different machine learning algorithms of BPANN, SVM, and ELM. Then, the forecasting outcomes of the different machine learning models were compared and analyzed. After that, by using the optimal machine learning model combined with Garson’s Algorithm, the sensitivity analysis was carried out to determine the relative significance of the input parameters on influencing the creep shear mechanical properties of the interfaces. In the end, the case study was conducted to verify the reliability for the results forecasted from the constructed machine learning model by comparing with the measured mechanical response of the interfaces in the physical tests. 

The main research outcomes are as follows: The hidden layer joint number has a considerable impact on the assessing precision of the BPANN and ELM models. Compared to SVM and ELM model, the BPANN model has a superior performance in forecasting the temperature-dependent long-term creep shear mechanical response of textured geomembrane–silica sand interfaces based on the assessment indexes of RMSE, R, WI, and MAPE. The sensitivity analysis indicates the influence of temperature on the creep shear mechanical properties of textured geomembrane–silica sand interfaces is the largest, followed by time, and creep shear pressure and normal pressure have relative low influence. 

For future research directions, it is advisable to consider the incorporation of additional factors into the modeling process, such as wet–dry cycling and the characterization of geomembranes (whether they are smooth or textured). Furthermore, the application of 3D printing technology can be explored to fabricate geomembranes with varying protrusion heights. Subsequent experiments can be conducted to gather extensive data, thus facilitating the expansion of the machine learning model’s database. These steps are essential in the development of a machine learning model with improved accuracy and enhanced generalization capabilities for the prediction of long-term creep mechanical properties at geomembrane–soil interfaces.

## Figures and Tables

**Table 1:** The properties of the test sample.

Textured geomembrane (Flat die extruded)	Thickness (mm)	2.0
	Textured height (mm)	0.26
	Fracturing strength (N/mm)	16.2
	Yield strength (N/mm)	22.3
	Yield elongation rate (%)	12.2
	Fracturing elongation rate (%)	120
	Puncture strength (N)	195
Silica sand	Particle size range (mm)	0.075~2
	Density (g/cm^3^)	1.50
	Optimum water content (%)	10
	Uniformity coefficient	3.327
	Curvature coefficient	0.3
	Median particle size (mm)	0.785

**Table 2:** Test scheme.

Test Sample	Normal Pressure (kPa)	Temperature (°C)	Creep Shear Stress
Silica sand–textured geomembrane interface	25, 50, 100	30, 60, 200	50%, 70%, and 90% of the monotonic interface peak shear strength

**Table 3:** The statistics.

	Parameters	Minimum Value	Maximum Value	Mean Value	Standard Deviation
Input	Normal pressure (kPa)	25	100	59	30.6
	Creep shear pressure	50% of the peak shear strength	90% of the peak shear strength	70% of the peak shear strength	20.39
	Time (minute)	0	8640	4320	650
	Temperature (°C)	30	200	95	20.72
Output	Creep shear displacement (mm)	0	47.20	24.50	6.40

**Table 4:** The test conditions and the corresponding input values.

Test Condition	Value	Input Value
Temperature	50 °C, 90 °C	50, 90
Time	2000 min, 4000 min	2000, 4000
Normal stress	60 kPa, 100 kPa	60, 100
Creep shear pressure	70% of the peak shear strength	70

## Data Availability

In this paper, all the data, models, and code used during the study appear in the submitted article.
